# Effect of a Traditional Chinese Herbal Medicine Formulation on Cell Survival and Apoptosis of MPP^+^-Treated MES 23.5 Dopaminergic Cells

**DOI:** 10.1155/2017/4764212

**Published:** 2017-05-18

**Authors:** Shuifen Ye, Ho Kee Koon, Wen Fan, Yihui Xu, Wei Wei, Chuanshan Xu, Jing Cai

**Affiliations:** ^1^Department of Integrative Medicine, Fujian University of Traditional Chinese Medicine, Fuzhou 350122, China; ^2^Longyan First Hospital Affiliated to Fujian Medical University, Longyan 364000, China; ^3^School of Chinese Medicine, Faculty of Medicine, The Chinese University of Hong Kong, Shatin, Hong Kong; ^4^Xiamen Haicang Hospital, Xiamen 361026, China; ^5^Second People's Hospital, Fujian University of Traditional Chinese Medicine, Fuzhou 350003, China; ^6^No. 477 Hospital of Chinese People's Liberation Army, Xiangyang 441000, China; ^7^Shenzhen Research Institute, The Chinese University of Hong Kong, Shenzhen 518057, China

## Abstract

Progressive degeneration of dopaminergic neurons in the substantia nigra (SN) is implicated in Parkinson's disease (PD). The efficacy of these currently used drugs is limited while traditional Chinese medicine (TCM) has been used in the management of neurodegenerative diseases for many years. This study was designed to evaluate the effect of a modified traditional Chinese herbal medicine decoction, Cong Rong Jing (CRJ), on cell survival and apoptosis of 1-methyl-4-phenylpyridinium- (MPP^+^-) treated MES23.5 dopaminergic cells. CRJ was prepared as a decoction from three Chinese herbs, namely,* Herba Cistanches*,* Herba Epimedii*, and* Rhizoma Polygonati*. We reported here that CRJ significantly enhanced the cell survival of MES23.5 cells after the exposure of MPP^+^ and inhibited the production of intracellular reactive oxygen species (ROS) induced by MPP^+^. CRJ also prevented the MPP^+^-treated MES23.5 cells from apoptosis by reducing the externalization of phosphatidylserine and enhancing the Bcl-2/Bax protein expression ratio. Signaling proteins such as JAK2, STAT3, and ERK1/2 were also involved in the action of CRJ. Taken together, these results provide a preliminary mechanism to support clinical application of the TCM formulation in PD and possibly other neurodegenerative diseases associated with ROS injury and apoptosis.

## 1. Introduction

Parkinson's disease (PD) is a neurodegenerative disorder due to the progressive and selective degeneration of dopaminergic neurons in the substantia nigra (SN), leading to the depletion of dopamine in striatum [1, 2]. Although the biochemical and molecular pathogenesis of the loss of dopaminergic neurons in PD has not yet been fully understood, it is believed that the pathogenesis is multifactorial which includes oxidative stress, mitochondrial dysfunction, and glutamate-mediated excitotoxicity and inflammation [3, 4]. Emerging evidence also shows that apoptotic pathways are probably involved in the death of dopaminergic neurons in PD [5, 6]. Prevention of the dopaminergic neurons from proceeding apoptosis would be useful in the treatment of PD.

Traditional Chinese medicine (TCM) has been shown to reduce the progression of the symptoms of PD for many years [7–10]. It exerts therapeutic effect in controlling the progression of the disease and reducing the dosage of dopamine for treatment [10]. We have previously tested five Chinese herbs (*Fructus Ligustri Lucidi, Herba Cistanches*,* Herba Epimedii Rhizoma Polygonati*, and* Semen Cuscutae*) with “kidney-tonifying” properties according to the theories of TCM and found that some of the herbs showed better neuroprotective effects in PD mouse model [11] and H_2_O_2_-injured MES23.5 cell model [12] as compared to selegiline, a monoamine oxidase inhibitor which is used to reduce early symptoms of PD. These herbs demonstrated differential neuroprotective effects by (1) increasing the neurotropic factors such as nerve growth factor (NGF), brain-derived neurotrophic factor (BDNF), and glial cell line-derived neurotrophic factor (GDNF) [11, 12], (2) reducing neuronal apoptosis through the inhibition of proapoptotic FasL and caspase-3 expression and enhancement of antiapoptotic Bcl-2 expression [11, 12], and (3) increasing tyrosine hydroxylase (TH) activity [11]. As the pathogenesis of PD is complex, it is expected that the herbal formulations may probably provide broader neuroprotective effects due to the multitargeted actions [13, 14]. Therefore, in the present study we selected three Chinese herbs (*Herba Cistanches*,* Herba Epimedii*, and* Rhizoma Polygonati*) from our previous findings to prepare a TCM formulated decoction, namely, Cong Rong Jing (CRJ), to further investigate the effect of the herbal formulation on cell survival and apoptosis of MPP^+^-treated MES23.5 cells.

## 2. Materials and Methods

### 2.1. Materials

Fetal bovine serum (FBS) and cell culture medium Dulbecco's modified Eagles' medium Nutrient Mixture-F12 (DMEM/F12) were purchased from Life Technologies (Waltham, MA, USA). AG490 (JAK2 inhibitor), PD98059 (ERK inhibitor), 3-(4,5-dimethylthiazol-2-yl)-2,5-diphenyltetrazolium bromide (MTT), 1-methyl-4-phenyl-pyridiniuiodide (MPP^+^), and 2′,7′-Dichlorofluorescin diacetate (DCFH-DA) were purchased from Sigma (St. Louis, MO, USA). Annexin V apoptosis detection kit was purchased from KeyGEN biotech (Nanjing, China). Antibodies of phospho-JAK2 (p-JAK2), JAK2, phospho-STAT3 (p-STAT3), STAT3, phospho-ERK1/2 (p-ERK1/2), ERK1/2, Bcl-2, Bax, and *β*-actin were purchased from Cell Signaling Technology (Beverly, MA, USA).

### 2.2. Preparation of the Aqueous Extract

The traditional Chinese medicinal herbs,* Herba Cistanches* (Rou Cong Rong),* Herba Epimedii* (Yin Yang Huo), and* Rhizoma Polygonati* (Huang Jing), were purchased from Fujian Pharmaceutical Co. Ltd. (Fuzhou, China) and were carefully authenticated by Laboratory of Pharmacognosy and Chinese Medicine according to the Chinese pharmacopoeia (The Pharmacopoeia Commission of People's Republic of China, 2005). To prepare CRJ aqueous extract,* Herba Cistanches* (50 g),* Herba Epimedii* (50 g), and* Rhizoma Polygonati* (90 g) were mixed and ground. The raw herbal powder was immersed in a total volume of 10 times (w/v) that of distilled water for 1 hour and then boiled for 2 hours. The solution was filtered and the filtrate was collected. The entire residue was collected and further boiled with a total volume of 8 times (w/v) that of distilled water for 2 hours. The solution was filtered and the two filtrates were combined, concentrated, and freeze-dried. The yield of the final dried extract was 25% (w/w) of the starting raw herbal materials and the resulting extract was stored at −20°C until used. The concentration of CRJ in this study was calculated according to the starting raw herbal materials. The stock solution CRJ (10 mg/mL) was prepared by dissolving CRJ in PBS, followed by sonication, sterilization at 100°C, and filtration.

### 2.3. Cell Culture

MES23.5 cells, which were originally established and developed by Dr. Weidong Le at Baylor College of Medicine, USA, were cultured as described in Li et al.'s report [15]. Briefly, MES23.5 cells were maintained in DMEM/F12 culture medium supplemented with 5% FBS (Life Technologies, Waltham, MA, USA), 1% L-glutamine (Sigma, St. Louis, MO, USA), 2% of 50x Sato's solution [12, 16], 100 U/mL of penicillin, and 0.1 mg/mL of streptomycin (Life Technologies, Waltham, MA, USA). The cells were maintained and incubated in a humidified 5% CO_2_ incubator at 37°C.

### 2.4. MPP^+^ and CRJ Treatment

MES23.5 cells were seeded in poly-D-lysine (PDL) coated 96-well plate at a density of 1 × 10^5^ cells per well. Different concentrations of MPP^+^ were administered to the cells for 24 or 48 hours to optimize the experimental condition. To evaluate the neuroprotective effect of CRJ, MPP^+^ containing medium was removed after 24 hours of incubation and then further treated with different concentrations of CRJ for 24 or 48 hours. The cells in the control were only treated by culture medium not containing CRJ and MPP^+^.

### 2.5. Cell Viability Assay

Cell viability was detected by MTT assay. After the indicated time of treatment, 20 *μ*L of MTT solution (5 mg/mL) was added to the cells and further incubated for 4 hours at dark environment. After that, the supernatant was removed and 150 *μ*L of DMSO was added to each well of the plate. The plate was further shaken for 10 min to dissolve the formazan crystal. Optical density of each well was measured by spectrophotometer (BIO-TEK ELX 800, BioTek Instruments, Inc., Vermont, USA). Freshly prepared DMEM/F12 culture medium was used as a negative control.

### 2.6. Detection of Intracellular ROS Production

Intracellular ROS level was examined using flow cytometry with H2DCF-DA staining as described by Wang et al. [17]. Briefly, the treated cells were washed with serum-free medium followed by incubation of DCFH-DA in the absence of light for 30 min at 37°C. Cells were then washed, centrifuged, and resuspended in PBS. The cells were analyzed by FACSVerse™ flow cytometer (Becton Dickinson, New Jersey, USA) with the excitation wavelength of 488 nm and the fluorescent signals were acquired by the FL-1 channel. Data were analyzed by the CellQuest software.

### 2.7. Apoptosis Detection

The percentage of apoptosis was detected using flow cytometry with Annexin V-fluorescein isothiocyanate (FITC) Apoptosis Detection Kit (NanJing KeyGen Biotech Co., Ltd, Nanjing, China) according to the manufacturer's instructions. Briefly, the treated cells were harvested and collected by EDTA-free trypsin. The action of trypsin was neutralized by serum-containing culture medium. At least 1 × 10^5^ cells were collected and washed once with cold PBS after the centrifugation. The cells were then suspended in 500 *μ*L binding buffer followed by the addition of staining (Annexin-V-FITC) reagent and propidium iodide (PI). After incubation in the dark at room temperature for 10 min, the cells were analyzed by BD FACSVerse flow cytometer (Becton Dickinson, New Jersey, USA). The results were further analyzed using Cell Quest software.

### 2.8. Western Blot Analysis

Control or treated MES23.5 cells were lysed in RIPA lysis buffer (Beyotime Co., Shanghai, China) containing 50 mM Tris-HCl (pH 7.4), 150 mM NaCl, 1% Triton X-100, 1% sodium deoxycholate, 0.1% sodium dodecyl sulfate (SDS), protease inhibitor (sodium orthovanadate, sodium fluoride, EDTA, and leupeptin), and phenylmethylsulfonyl fluoride (PMSF, 1 mM). Protein concentration of the cell lysates was measured by BCA assay. Cell lysates (50 *μ*g) were then loaded and separated by 10% SDS gel and then transferred to polyvinylidene difluoride (PVDF) membrane. Blots were probed with p-JAK2, JAK2, p-STAT3, STAT3, p-ERK1/2, ERK1/2, Bcl-2, Bax, and *β*-actin (1 : 1000) at 4°C overnight. After washing with TBST, the membrane was incubated with an appropriately diluted secondary antibody (1 : 5000) conjugated with horseradish peroxidase for 1 hour at room temperature. Chemiluminescence was detected using the Western blotting substrate (ECL) and visualized on an X-ray film. ImageJ software was used to measure the densitometry of bands generated from Western blot analysis.

### 2.9. Statistical Analysis

Each experiment was performed at least three times and data were expressed as mean ± SEM and analyzed using Graphpad prism v.6.0. Time course changes in protein expression were analyzed by unpaired Student's* t*-test. One-way analysis of variance (ANOVA) followed by Turkey's multiple comparison post hoc test was used to compare the differences between groups. A value of *P* < 0.05 was considered to be statistically significant. 

## 3. Results

### 3.1. CRJ Enhanced Cell Survival of MPP^+^-Treated MES23.5 Cells

**Figure 1:**
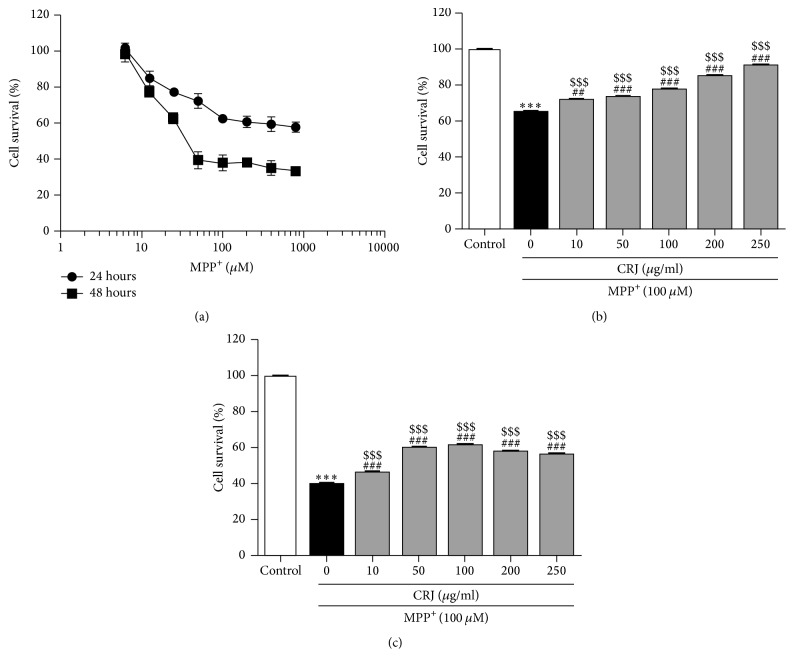
Effect of CRJ on the cell survival in MPP^+^-treated MES23.5 dopaminergic neurons. (a) Exposure of MPP^+^ alone for 24 or 48 hours resulted in the decrease of cell survival in MES23.5 cells. Posttreatment of different concentration of CRJ for (b) 24 or (c) 48 hours in MPP^+^-treated MES23.5 cells enhanced the cell survival as compared to the MPP^+^ treatment group without CRJ treatment. Data were represented as mean ± SEM in three independent experiments. ^*∗∗∗*^*P* < 0.001, MPP^+^-treated cells as compared to control. ^##^*P* < 0.01, ^###^*P* < 0.001, CRJ + MPP^+^ groups as compared to MPP^+^-treated cells. ^$$$^*P* < 0.001, CRJ + MPP^+^ groups as compared to control.

The concentrations of MPP^+^ and CRJ for the treatment of MES23.5 dopaminergic cells were optimized for the present study using MTT assay. CRJ treatment alone showed no significant cytotoxicity effect on MES23.5 cells at the concentration of 250 *μ*g/mL. MPP^+^ treatment demonstrated dose- and time-dependent cytotoxicity to MES23.5 cells at the concentrations of 12.5 to 800 *μ*M (Figure 1(a)). The treatment of different concentrations of CRJ (100, 200, and 250 *μ*g/mL) significantly increased the cell survival of MPP^+^-treated MES23.5 cells from 65% to 91% and from 40% to 56% at 24 hours (Figure 1(b)) and 48 hours (Figure 1(c)), respectively (*P* < 0.001).

### 3.2. CRJ Reduced ROS Production in MES23.5 Cells after MPP^+^ Treatment

**Figure 2:**
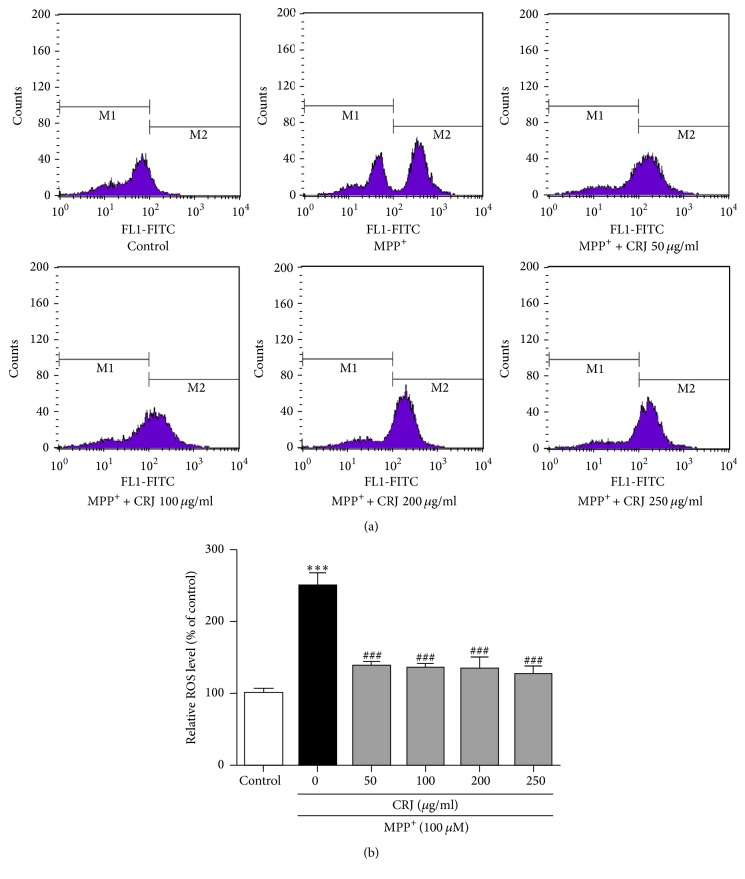
Detection of ROS in MES23.5 cells using flow cytometric analysis. MES23.5 cells were treated with MPP^+^ (100 *μ*M) for 24 hours, followed by the posttreatment of CRJ for another 24 hours. MPP^+^ (100 *μ*M) increased the production of ROS in MES23.5 cells. Posttreatment of CRJ resulted in the decrease of ROS production in MPP^+^-treated MES23.5 cells. Fluorescence intensity of control group was set as 100%. Data were represented as mean ± SEM in three independent experiments. ^*∗∗∗*^*P* < 0.001, MPP^+^-treated cells as compared to control. ^###^*P* < 0.001, CRJ + MPP^+^ groups as compared to MPP^+^-treated cells.

MPP^+^ is well known to induce the production of ROS and cause neurotoxicity [18, 19]. To evaluate whether the rescue of MPP^+^-treated MES23.5 cells by CRJ is associated with the level of intracellular ROS, an indirect measurement of ROS using fluorescence method was adopted.  Figure 2 shows a significant increase in ROS level in MPP^+^-treated MES23.5 cells as compared to the control (*P* < 0.001). However, the treatment of CRJ significantly reduced the generation of intracellular ROS level after MPP^+^ treatment, as compared to MPP^+^-treated cells alone (*P* < 0.001). This indicated that CRJ may exhibit the neuroprotective effect in MPP^+^-treated MES23.5 via the removal of intracellular ROS.

### 3.3. CRJ Reduced MPP^+^-Induced Apoptosis in MES23.5 Cells

**Figure 3:**
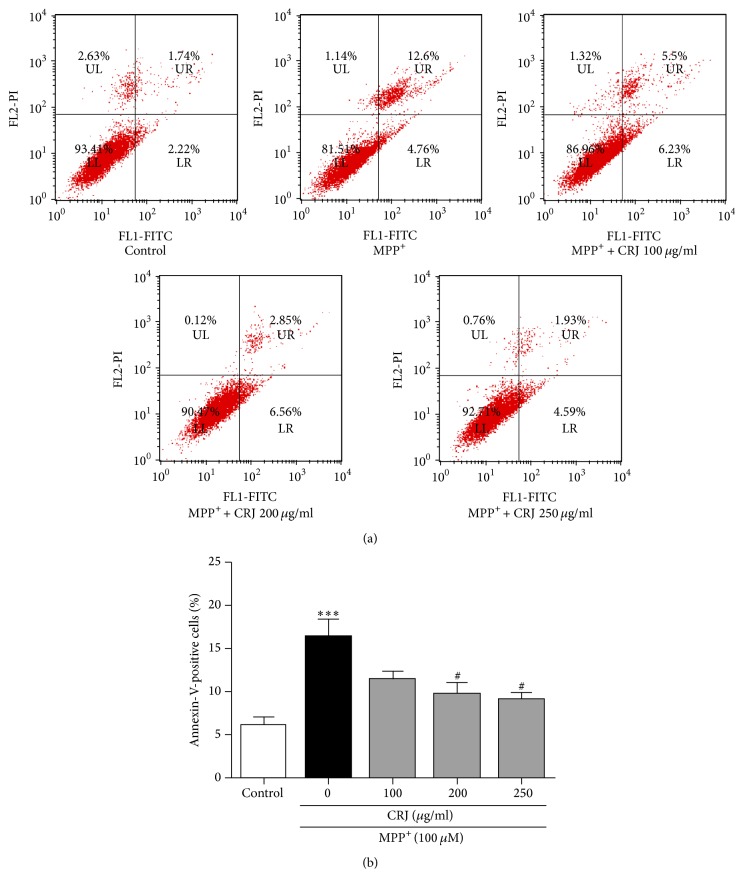
Percentage of Annexin-V-positive cells was analyzed by flow cytometry. MES23.5 cells were treated as described in Figure 2. MPP^+^ (100 *μ*M) increased the percentage of Annexin-V-positive MES23.5 cells. Posttreatment of CRJ resulted in the decrease of Annexin-V-positive MES23.5 cells after the exposure of MPP^+^. Data were represented as mean ± SEM in three independent experiments. ^*∗∗∗*^*P* < 0.001, MPP^+^-treated cells as compared to control. ^#^*P* < 0.05, CRJ + MPP^+^ groups as compared to MPP^+^-treated cells.

Phosphatidylserine is a phospholipid located at inner plasma membrane. During the early apoptosis, phosphatidylserine will translocate to the outer plasma membrane [20]. The externalization of phosphatidylserine indicates the early event of apoptosis and could be revealed by the binding of Annexin V-FITC. PI counterstain was used to detect cells undergoing necrosis or late apoptosis. In this study, MPP^+^-treated MES23.5 cells were positively stained with Annexin V-FITC after 24 hours, indicating that MPP^+^ triggered the apoptotic process. In the presence of CRJ, the percentage of Annexin V-FITC positively stained cells significantly decreased (*P* < 0.05) in the dose-dependent manner (Figure 3), indicating that CRJ treatment reduced MPP^+^-induced apoptosis in MES23.5 cells.

### 3.4. CRJ Increased the Ratio of Bcl-2/Bax in the MPP^+^-Treated MES23.5 Cells

**Figure 4:**
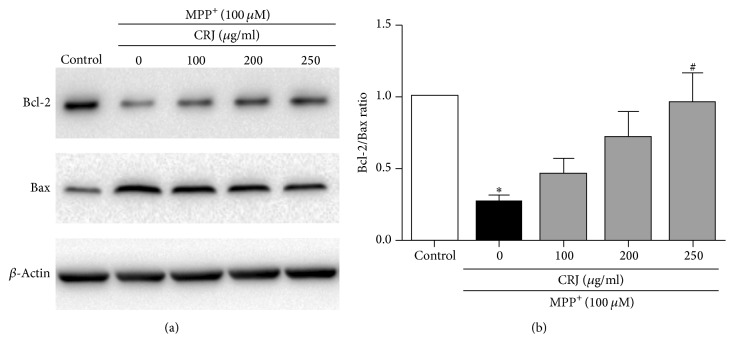
Effects of CRJ on the Bcl-2/Bax ratio in MPP^+^-treated MES23.5 cells. MES23.5 cells were treated as described in Figure 2. (a) Expression of antiapoptotic (Bcl-2) and proapoptotic (Bax) proteins. *β*-Actin was used as protein loading control. (b) The Bcl-2/Bax ratio was determined by densitometric analysis of bands from Western blot. Data were represented as mean ± SEM in three independent experiments. ^*∗*^*P* < 0.05, compared to control. ^#^*P* < 0.05, compared to MPP^+^-treated cells.

To further confirm the mode of cell protection of CRJ in MPP^+^-induced neurocytotoxicity in MES23.5 cells, the expression of the ratio of Bcl-2 and Bax proteins was determined by Western blot analysis. It is well known that Bcl-2 is the antiapoptotic protein while Bax is the proapoptotic protein [18]. The decrease of the ratio of Bcl-2/Bax could favour the process of intrinsic mitochondria-mediated apoptosis [21, 22]. In this study, MPP^+^ downregulated the protein expression of Bcl-2 while upregulating the expression of Bax in MES23.5 cells (Figure 4(a)). The treatment of CRJ increased the expression of Bcl-2 while decreasing the expression of Bax in MPP^+^-treated MES23.5 cells in the dose-dependent manner, resulting in a significant increase (*P* < 0.05) of the overall ratio of Bcl-2/Bax (Figure 4(b)).

### 3.5. Modulation of the Expression of JAK2/STAT3 and ERK1/2 by CRJ in Untreated or MPP^+^-Treated MES23.5 Cells

**Figure 5:**
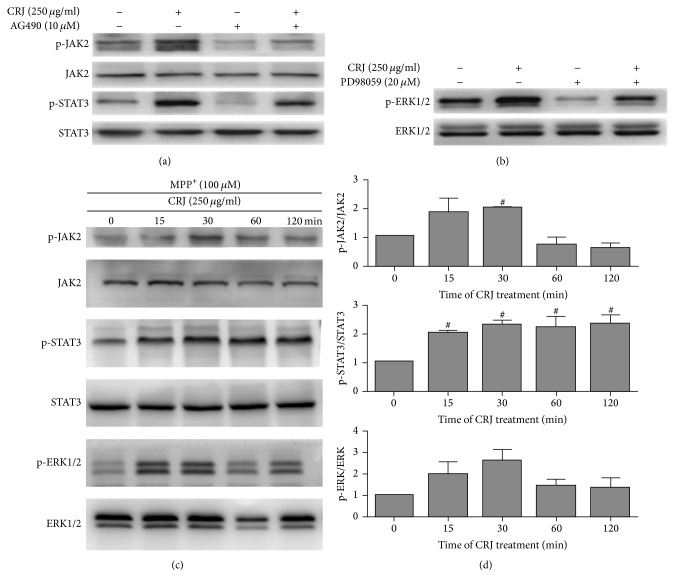
Protein expression of p-JAK2, p-STAT3, and p-ERK1/2 in MES23.5 cells and MPP^+^-treated MES23.5 cells. ((a) and (b)) MES23.5 cells were pretreated with AG490 (JAK inhibitor) or PD98059 (ERK1/2 inhibitor) for 1 hour, followed by CRJ (250 *μ*g/mL) incubation for 24 hours. CRJ alone enhanced the expression of (a) p-JAK2 and p-STAT3 and (b) p-ERK1/2 in MES23.5 cells. The effect could be inhibited by the corresponding pharmacological inhibitors. ((c) and (d)) Time courses of the changes of protein levels of p-JAK2, p-STAT3, and p-ERK1/2 in MPP^+^-treated MES23.5 cells after the posttreatment of CRJ (250 *μ*g/mL). Densitometric analysis of protein expression was determined. Data were represented as mean ± SEM in three independent experiments. Total form of each of the phosphorylated proteins was used as protein loading control. ^#^*P* < 0.05, compared to MPP+-treated cells 0 min after the posttreatment of CRJ.

JAK2/STAT3 and/or survival signaling pathway have been reported to associate with the expression of Bcl-2 and Bax [23, 24]. Therefore, we attempted to further investigate the effect of CRJ treatment on the expression of JAK2/STAT3 and ERK1/2 signaling proteins in our model. Untreated MES23.5 cells were pretreated with either AG490 (JAK2 inhibitor) or PD98059 (ERK1/2 inhibitor) for 1 hour, followed by treatment of CRJ for 24 hours. Total cell lysates were then collected and the phosphorylation states of JAK2 (p-JAK2), STAT3 (p-STAT3), and ERK1/2 (p-ERK1/2) were determined by Western blot analysis (Figures 5(a) and 5(b)). The results showed that p-JAK2, p-STAT3, and p-ERK1/2 were found to be expressed in the untreated MES23.5 cells. Treatment of CRJ could further increase the expression of p-JAK2, p-STAT3, and p-ERK1/2 in MES23.5 cells. The activation of p-JAK2, p-STAT3, and p-ERK1/2 by CRJ was partially inhibited by AG490 and PD98059, respectively. The results suggested that CRJ was involved in the upregulation of the expression of p-JAK2, p-STAT3, and p-ERK1/2 signaling proteins.

We further tested the effect of CRJ treatment on the p-JAK2, p-STAT3, and p-ERK1/2 signaling proteins in MES23.5 cells after 24-hour treatment with MPP^+^ (100 *μ*M). We found that the treatment of CRJ (250 *μ*g/mL) in the first 30 min and 60 min after MPP^+^ treatment significantly activated the expression of p-JAK2 and p-STAT3 (*P* < 0.05) and slightly increased p-ERK1/2 (*P* = 0.05) (Figures 5(c) and 5(d)). This indicated that further upregulation of the p-JAK2, p-STAT3, and probably p-ERK1/2 protein expressions in MPP^+^-treated MES23.5 cells would be associated with the treatment of CRJ.

## 4. Discussion

Currently, dopamine replacement therapy is the first-line clinical management to control the motor symptoms in PD patients. However, the treatment could only be maintained for few years due to the development of the end-of-dose and on-off phenomenon [25]. Neuroprotection has emerged as one of the main interests in PD researches [26]. Identification of drugs that lead to preventing the dopaminergic neurons from apoptosis and oxidative stress may probably help reduce the dosage and side effects of dopamine replacement therapy. Accelerating evidences show that some active ingredients of* Cistanches Herba* and* Herba Epimedii* such as phenylethanoid glycosides, echinacoside, and icariin exhibit antioxidant and neuroprotective activities [27–29]. Our previous studies showed that the decoction of different “kidney-tonifying” Chinese herbs regulated the expression of apoptotic-related factors and also neurotrophic factors in PD cell and animal models [11, 12]. Since the pathological pathways of PD are multifactorial and complex, the neuroprotective actions of a single herbal medicine are limited. For example, in the PD mouse model,* Herba Epimedii* prevented the loss of TH activity but not Bcl-2, while* Rhizoma Polygonati* was able to reduce the expression of apoptosis-promoting factors in the model but had no effect on the TH activity [11]. Therefore, in this study, we aimed at evaluating the therapeutic actions of CRJ, a TCM formulation comprising three selected Chinese herbal medicines (*Herba Cistanches*,* Herba Epimedii*, and* Rhizoma Polygonati*) instead of single Chinese herbs, in a MPP^+^-injured dopaminergic cell model. We observed that CRJ could exhibit multiple significant protective effects.

MPP^+^ has been demonstrated as a neurotoxin that inhibits complex I of the mitochondrial electron-transport chain, which leads to oxidative stress and mitochondrial dysfunction in MES23.5 cells and other neuronal cell types [30–32]. In this study, CRJ was found to partially abolish the ROS in MES23.5 cells after MPP^+^ treatment. It is a crucial observation as the dopaminergic neurons keep generating ROS including hydrogen peroxide and hydroxyl radicals during the dopamine metabolism [33, 34]. It is believed that the dopaminergic neurons would be less vulnerable to oxidative injury in the presence of CRJ. Another common consequence of mitochondrial dysfunction would be the initiation of intrinsic mitochondrion-mediated apoptotic pathway. The externalization of phosphatidylserine at inner plasma membrane and the alternation of the balance between antiapoptotic and proapoptotic Bcl-2 family proteins would finally lead to the downstream cascades of intrinsic apoptotic cell death [20]. Our study showed that CRJ might play a central role in the prevention of apoptotic cell death induced by MPP^+^ through the modulation of Bcl-2 and Bax proteins and prevention of the externalization of phosphatidylserine.

JAK/STAT signaling pathway has been recognized as a conserved signaling pathway involved in both physiological and pathological cellular events such as proliferation [35], differentiation [36], and survival [37, 38]. Blockage of the JAK2/STAT3 pathway using pharmacological inhibitor AG490 has been shown to reduce the neuronal survival [38] and abolish the neuroprotective effect of neuroprotectants [39, 40]. In the present study, significant upregulation of p-JAK2 and p-STAT3 was observed. The neuroprotective effect of CRJ in MPP^+^-treated MES23.5 cells was likely associated with the phosphorylation of JAK2 and STAT3, resulting in the reduction of apoptosis.

ERK is another important signaling pathway which mediates cell survival. Many neuroprotectants are found to protect neuronal cell death via the activation of ERK signaling pathway. Activation of ERK pathways by long-term administration of valproic acid (VPA) enhanced neurite growth, cell survival in SH-SY5Y cells [41], ERK-dependent gene expression of Bcl-2, and neurogenesis in mice embryonic cortical neurons and adult hippocampus [42]. Ginsenoside Rb1 prevents MPP^+^-induced apoptosis in PC12 cells through the activation of ERK/Akt pathways and inhibition of SAPK/JNK and p38 MAPK pathways [43]. In this study, the treatment of CRJ did not significantly increase the expression of ERK in MPP^+^-treated MES23.5 cells but it upregulated the expression of ERK in untreated MES23.5 cells and also showed nonsignificant trend of transient activation of ERK in MPP^+^-treated cells. It may be implicated that CRJ would probably induce the cell survival pathways in MES23.5 cells, leading to the reduction of MPP^+^-induced apoptosis.

## 5. Conclusion

In summary, the present study has demonstrated significant protective actions of a TCM formulation, CRJ, which includes the Chinese herbs* Herba Cistanches*,* Herba Epimedii*, and* Rhizoma Polygonati*, on MPP^+^-treated MES23.5 dopaminergic cells. It is believed that the neuroprotection of the Chinese herbal formulation is “multitargeted.” Based on the preclinical findings in this study, it is speculated that the CRJ formulation would be a potential candidate for the management of PD or possibly other neurodegenerative diseases that involve oxidative injury and neuronal apoptosis.

## References

[1] Bohnen N. I., Albin R. L. (2011). The cholinergic system and Parkinson disease. *Behavioural Brain Research*.

[2] Calabresi P., Picconi B., Parnetti L., Di Filippo M. (2006). A convergent model for cognitive dysfunctions in Parkinson's disease: the critical dopamine-acetylcholine synaptic balance. *Lancet Neurology*.

[3] Martinez-Castrillo J. C., Vela L., Del Val J., Alonso-Canovas A. (2011). Nonmotor disorders and their correlation with dopamine: can they be treated by currently available methods?. *Neurologist*.

[4] Mullin S., Schapira A. H. V. (2015). Pathogenic mechanisms of neurodegeneration in parkinson disease. *Neurologic Clinics*.

[5] Tatton W. G., Chalmers-Redman R., Brown D., Tatton N. (2003). Apoptosis in Parkinson's disease: signals for neuronal degradation. *Annals of Neurology*.

[6] Singh S., Dikshit M. (2007). Apoptotic neuronal death in Parkinson's disease: involvement of nitric oxide. *Brain Research Reviews*.

[7] Chen L. W., Wang Y. Q., Wei L. C., Shi M., Chan Y. S. (2007). Chinese herbs and herbal extracts for neuroprotection of dopaminergic neurons and potential therapeutic treatment of Parkinson's disease. *CNS and Neurological Disorders: Drug Targets*.

[8] Li M., Yang M.-H., Liu Y., Luo X.-D., Chen J.-Z., Shi H.-J. (2015). Analysis of clinical evaluation of response to treatment of Parkinson’s disease with integrated Chinese and Western medicine therapy. *Chinese Journal of Integrative Medicine*.

[9] Zhang J., Ma Y.-Z., Shen X.-M. (2013). Evaluation on the efficacy and safety of Chinese herbal medication Xifeng Dingchan Pill in treating Parkinson's disease: Study protocol of a multicenter, open-label, randomized active-controlled trial. *Journal of Chinese Integrative Medicine*.

[10] Li Q., Zhao D., Bezard E. (2006). Traditional Chinese medicine for Parkinson's disease: a review of Chinese literature. *Behavioural Pharmacology*.

[11] Cai J., Tian Y., Lin R., Chen X., Liu Z., Xie J. (2012). Protective effects of kidney-tonifying Chinese herbal preparation on substantia nigra neurons in a mouse model of Parkinson's disease. *Neural Regeneration Research*.

[12] Lin S., Ye S., Huang J. (2013). How do Chinese medicines that tonify the kidney inhibit dopaminergic neuron apoptosis?. *Neural Regeneration Research*.

[13] Song J.-X., Sze S. C.-W., Ng T.-B. (2012). Anti-Parkinsonian drug discovery from herbal medicines: what have we got from neurotoxic models?. *Journal of Ethnopharmacology*.

[14] Li X.-Z., Zhang S.-N., Liu S.-M., Lu F. (2013). Recent advances in herbal medicines treating Parkinson's disease. *Fitoterapia*.

[15] Li X.-P., Xie W.-J., Zhang Z., Kansara S., Jankovic J., Le W.-D. (2012). A mechanistic study of proteasome inhibition-induced iron misregulation in dopamine neuron degeneration. *NeuroSignals*.

[16] Bottenstein J. E., Sato G. H. (1979). Growth of a rat neuroblastoma cell line in serum-free supplemented medium. *Proceedings of the National Academy of Sciences of the United States of America*.

[17] Wang J., Du X.-X., Jiang H., Xie J.-X. (2009). Curcumin attenuates 6-hydroxydopamine-induced cytotoxicity by anti-oxidation and nuclear factor-kappaB modulation in MES23.5 cells. *Biochemical Pharmacology*.

[18] Lotharius J., Dugan L. L., O'Malley K. L. (1999). Distinct mechanisms underlie neurotoxin-mediated cell death in cultured dopaminergic neurons. *The Journal of Neuroscience*.

[19] Simonian N. A., Coyle J. T. (1996). Oxidative stress in neurodegenerative diseases. *Annual Review of Pharmacology and Toxicology*.

[20] Elmore S. (2007). Apoptosis: a review of programmed cell death. *Toxicologic Pathology*.

[21] Shi Y. (2001). A structural view of mitochondria-mediated apoptosis. *Nature Structural & Molecular Biology*.

[22] Youle R. J., Strasser A. (2008). The BCL-2 protein family: opposing activities that mediate cell death. *Nature Reviews Molecular Cell Biology*.

[23] Badr G., Mohany M., Abu-Tarboush F. (2011). Thymoquinone decreases F-actin polymerization and the proliferation of human multiple myeloma cells by suppressing STAT3 phosphorylation and Bcl2/Bcl-XL expression. *Lipids in Health and Disease*.

[24] Wang C. X., Song J. H., Song D. K., Yong V. W., Shuaib A., Hao C. (2006). Cyclin-dependent kinase-5 prevents neuronal apoptosis through ERK-mediated upregulation of Bcl-2. *Cell Death and Differentiation*.

[25] Sweet R. D., McDowell F. H. (1974). Plasma dopa concentrations and the “on-off” effect after chronic treatment of parkinson’s disease. *Neurology*.

[26] Boll M.-C., Alcaraz-Zubeldia M., Rios C. (2011). Medical management of parkinson's disease: focus on neuroprotection. *Current Neuropharmacology*.

[27] Li Z., Lin H., Gu L., Gao J., Tzeng C.-M. (2016). Herba Cistanche (Rou Cong-Rong): One of the best pharmaceutical gifts of traditional Chinese medicine. *Frontiers in Pharmacology*.

[28] Chen M., Wu J., Luo Q. (2016). The anticancer properties of herba epimedii and its main bioactive componentsicariin and icariside II. *Nutrients*.

[29] Xu A. L., Jiang M. C., Chen X. H., Chen W. F. (2016). Icariin protects against MPP(+)-induced neurotoxicity in MES23.5 cells. *Sheng Li Xue Bao*.

[30] Jung T. W., Lee J. Y., Shim W. S. (2007). Rosiglitazone protects human neuroblastoma SH-SY5Y cells against MPP+ induced cytotoxicity via inhibition of mitochondrial dysfunction and ROS production. *Journal of the Neurological Sciences*.

[31] Liu W.-B., Zhou J., Qu Y. (2010). Neuroprotective effect of osthole on MPP^+^-induced cytotoxicity in PC12 cells via inhibition of mitochondrial dysfunction and ROS production. *Neurochemistry International*.

[32] Xu H., Jiang H., Wang J., Xie J. (2010). Rg1 protects the MPP+-treated MES23.5 cells via attenuating DMT1 up-regulation and cellular iron uptake. *Neuropharmacology*.

[33] Zhang K., Ma Z., Wang J., Xie A., Xie J. (2011). Myricetin attenuated MPP^+^-induced cytotoxicity by anti-oxidation and inhibition of MKK4 and JNK activation in MES23.5 cells. *Neuropharmacology*.

[34] Lotharius J., Brundin P. (2002). Pathogenesis of Parkinson's disease: dopamine, vesicles and alpha-synuclein. *Nature Reviews. Neuroscience*.

[35] Tsuda M., Kohro Y., Yano T. (2011). JAK-STAT3 pathway regulates spinal astrocyte proliferation and neuropathic pain maintenance in rats. *Brain*.

[36] Kim Y. H., Chung J.-I., Woo H. G. (2010). Differential regulation of proliferation and differentiation in neural precursor cells by the Jak pathway. *Stem Cells*.

[37] Monroe R. K., Halvorsen S. W. (2006). Cadmium blocks receptor-mediated Jak/STAT signaling in neurons by oxidative stress. *Free Radical Biology and Medicine*.

[38] Yadav A., Kalita A., Dhillon S., Banerjee K. (2005). JAK/STAT3 pathway is involved in survival of neurons in response to insulin-like growth factor and negatively regulated by suppressor of cytokine signaling-3. *Journal of Biological Chemistry*.

[39] Yu H. M., Zhi J. L., Cui Y. (2006). Role of the JAK-STAT pathway in protection of hydrogen peroxide preconditioning against apoptosis induced by oxidative stress in PC12 cells. *Apoptosis*.

[40] Kretz A., Happold C. J., Marticke J. K., Isenmann S. (2005). Erythropoietin promotes regeneration of adult CNS neurons via Jak2/Stat3 and PI3K/AKT pathway activation. *Molecular and Cellular Neuroscience*.

[41] Yuan P.-X., Huang L.-D., Jiang Y.-M., Gutkind J. S., Manji H. K., Chen G. (2001). The mood stabilizer valproic acid activates mitogen-activated protein kinases and promotes neurite growth. *Journal of Biological Chemistry*.

[42] Hao Y., Creson T., Zhang L. (2004). Mood stabilizer valproate promotes ERK pathway-dependent cortical neuronal growth and neurogenesis. *Journal of Neuroscience*.

[43] Hashimoto R., Yu J., Koizumi H., Ouchi Y., Okabe T. (2012). Ginsenoside Rb1 prevents MPP^+^-induced apoptosis in PC12 cells by stimulating estrogen receptors with consequent activation of ERK1/2, Akt and inhibition of SAPK/JNK, p38 MAPK. *Evidence-Based Complementary and Alternative Medicine*.

